# Biogeographic Patterns and Community Assembly Processes of Bacterioplankton and Potential Pathogens in Subtropical Estuaries in China

**DOI:** 10.1128/spectrum.03683-22

**Published:** 2022-12-12

**Authors:** Wenjian Chen, Shilei Sang, Liyi Shao, Yusen Li, Tongzhou Li, Lihong Gan, Li Liu, Dapeng Wang, Lei Zhou

**Affiliations:** a College of Marine Sciences, South China Agricultural University, Guangzhou, China; b State Environmental Protection Key Laboratory of Environmental Pollution Health Risk Assessment, South China Institute of Environmental Sciences, MEE, Guangzhou, China; c Guangxi Key Laboratory of Aquatic Genetic Breeding and Healthy Aquaculture, Guangxi Academy of Fishery Sciences, Nanning, Guangxi, China; Fujian Agruculture and Forestry University

**Keywords:** bacterioplankton, potential pathogen, community assembly, biogeographic patterns

## Abstract

Microbial communities in coastal waters are diverse and dynamic and play important roles in ecosystem functions and services. Despite the ecological impact of bacterioplankton or pathogens, little is known about whether bacterioplankton and pathogen communities exhibit similar patterns. Here, using 16S RNA gene amplicon sequencing, the geographic patterns and assembly processes of bacterioplankton and pathogen communities in 30 subtropical estuaries were studied. Results showed that the estuarine bacterioplankton communities mainly consisted of *Proteobacteria* (49.06%), *Actinobacteria* (17.62%), and *Bacteroidetes* (16.33%), among which 31 pathogen genera (186 amplicon sequence variants [ASVs]) were identified. Under the influence of salinity, bacterioplankton and pathogens showed similar biogeographic patterns. Redundancy and correlation analyses indicated that the bacterioplankton communities were strongly correlated with estuarine environmental factors, but potential pathogens were less influenced. Co-occurrence network analysis revealed a close relationship between bacterioplankton and potential pathogens, with two pathogens identified as connectors (i.e., ASV340 [Clostridium perfringens] and ASV1624 [Brevundimonas diminuta]), implying potential impacts of pathogens on structure, function, and stability of estuarine bacterioplankton communities. Null-model analysis revealed that deterministic processes (heterogeneous selection) dominated bacterioplankton community assembly, while stochastic processes (undominated effect) shaped the potential pathogen community. Our findings illustrate the biogeographic patterns and community assembly mechanisms of bacterioplankton and pathogens in estuaries, which should provide guidance and a reference for the control of potential pathogenic bacteria.

**IMPORTANCE** Bacterioplankton play an important role in estuarine ecosystem functions and services; however, potentially pathogenic bacteria may exhibit infectivity and pose a serious threat to environmental and human health. In this study, geographic patterns and assembly processes of bacterioplankton communities in 30 subtropical estuaries were explored, and potential pathogenic bacteria in the estuaries were detected and profiled. Our results demonstrate here that bacterioplankton and pathogens show similar biogeographic patterns under the influence of salinity. Interestingly, heterogeneous selection dominated bacterioplankton assembly, while stochasticity dominated pathogen assembly. This study provides important information for future risk assessment of potential pathogenic bacteria as well as management in estuarine ecosystems.

## INTRODUCTION

Estuaries connect terrestrial, freshwater, and marine ecosystems, with strong land-sea interactions and unique physical and chemical properties (1). As a result, the biological communities in estuaries are extremely complex and include a wide variety of bacteria, phytoplankton, zooplankton, and freshwater and marine plants and animals (2). However, due to the highly dynamic and complex environment, estuaries are particularly vulnerable to pressure and disturbance from natural processes and anthropogenic activities over time.

Bacteria are an extremely diverse group of organisms that play vital roles in biogeochemical cycling and aquatic ecosystem stability (3, 4). Heterotrophic bacteria participate in nitrification and ammoniation when consuming oxygen to degrade organic matter (5). Bacteria also absorb carbon dissolved in estuaries and fixed in organic matter particles (6, 7). In estuaries with low primary productivity, allochthonous carbon can become an important supporting factor for the food web through secondary bacterial production and energy and carbon transfer at higher trophic levels (8, 9). Furthermore, *in situ* bacteria may be potential biomarkers for monitoring standard pollutants and assessing environmental stressors in water (10). However, potentially pathogenic bacteria may exhibit infectivity and pose a serious threat to environmental and human health (11–13). For example, the fast-growing pathogen Clostridium perfringens can release more than 20 different toxins and is a member of the gastrointestinal community in both diseased and nondiseased humans and animals (14). Arcobacter cryaerophilus and other *Arcobacter* species have been identified as potential human pathogens and isolated in animals with gastritis, enteritis, mastitis, spontaneous abortion, and septicemia (15, 16). Many epidemic diseases (e.g., gastroenteritis, meningitis, pneumonia, septicemia, and zoonoses) are caused by pathogen-contaminated water (12, 13, 17, 18). Although pathogenic infections caused by contaminated water remain the most common environmental problem worldwide, little is known about the diversity, distribution, and environmental influences of pathogenic bacteria at the community level, especially in estuarine environments. Therefore, a full understanding of the biogeography and assembly mechanisms of bacterioplankton and pathogen communities is important.

The processes of bacterial community assembly and diversity are ecologically significant in water environments. Understanding the relative significance of deterministic and stochastic processes in assembly is vital for exploring how communities adapt to environmental changes and clarifying ecological processes (19–23). The neutral theory asserts that stochastic processes (e.g., birth, death, colonization, extinction, and speciation) dominate the formation of bacterial diversity (24–26). In contrast, niche theory predicts that bacterioplankton communities are largely controlled by abiotic (e.g., salinity and chlorophyll *a* [Chl*a*]) and biotic (e.g., competition, predation, and reciprocity) deterministic factors due to different preferences and suitability of microorganisms for habitats (27, 28). Coastal ecosystems are subject natural stresses, such as sudden changes in salinity concentrations (29), as well as chemical pollution and eutrophication (30). Deterministic processes are the main ecological mechanism underlying estuarine bacterial community assembly in previous studies (31–33). However, given the differences in environmental responses and dispersal abilities, it remains unclear whether bacterial and pathogen communities exhibit similar patterns.

We used 16S rRNA gene amplicon sequencing to profile the compositions of the bacterioplankton and potential pathogen communities in 30 subtropical estuaries in southern China. The purposes of this study were to (i) compare the biogeographic patterns of the bacterioplankton and potential pathogen communities, (ii) gain insight into the factors affecting the bacterioplankton and potential pathogenic communities, and (iii) explore the relative significance of stochastic and deterministic processes in driving the assembly of the bacterioplankton and potential pathogen communities.

## RESULTS

### Bacterioplankton and potential pathogen composition.

**FIG 1:**
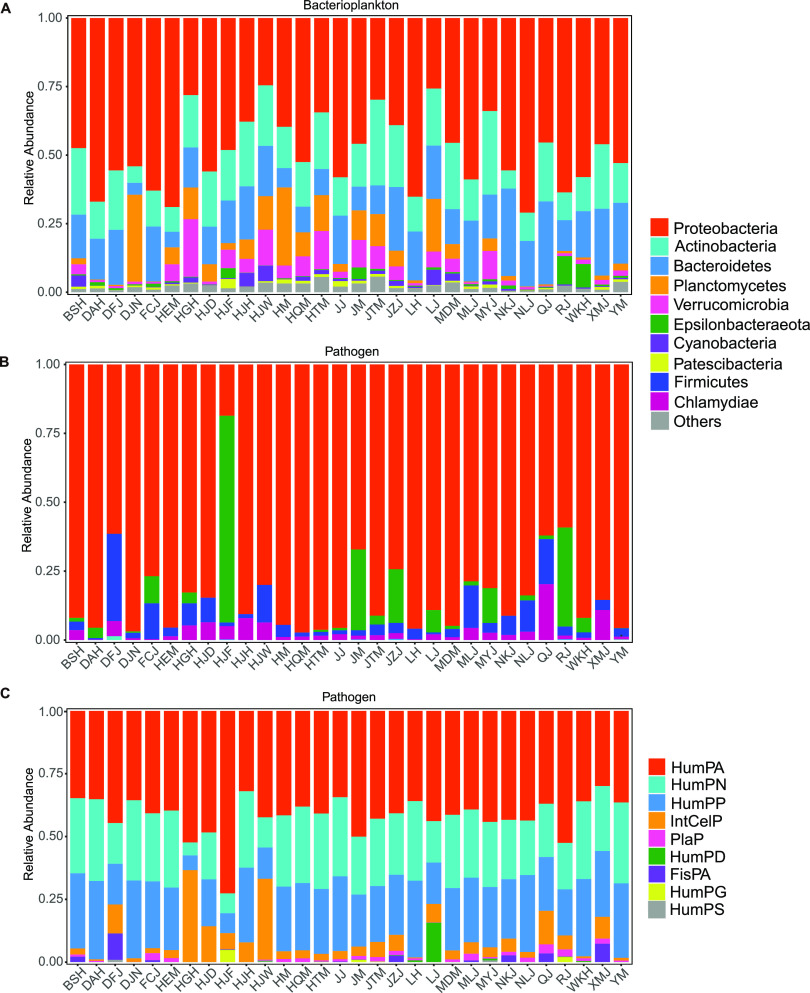
Composition and functional distribution of bacterioplankton and potential pathogen communities in 30 subtropical estuaries. (A and B) Bacterioplankton (A) and potential pathogen (B) abundance of sequences at the phylum level; (C) assignment of sampling points to functional groups.

At the phylum level, the main bacterioplankton included *Proteobacteria* (49.06%), *Actinobacteria* (17.62%), *Bacteroidetes* (16.33%), *Planctomycetes* (6.68%), *Verrucomicrobia* (4.77%), *Epsilonbacteraeota* (1.33%), *Cyanobacteria* (1.29%), and *Patescibacteria* (0.74%) (Fig. 1A). At the genus level, dominant bacterioplankton included *Fluviicola* and *Flavobacterium*, which constituted 18.06% and 17.66% of the total number of sequences, respectively. We then extracted groups of potential pathogens and parasites that may carry risk for humans, animals, or plants. In total, 186 amplicon sequence variants (ASVs) and 97,819 sequences were classified into nine potential pathogenic groups, including intracellular parasites (IntCelP [129 ASVs]), human potential pathogens—all (HumPA [64 ASVs]), human potential pathogens—pneumonia (HumPP [8 ASVs]), human potential pathogens—nosocomial (HumPN [6 ASVs]), plant potential pathogens (PlaP [5 ASVs]), human potential pathogens—diarrhea (HumPD [5 ASVs]), human potential pathogens—septicemia (HumPS [2 ASVs]), fish parasites (FisPA [2 ASVs]), and human potential pathogens—gastroenteritis (HumPG [1 ASV]). Among them, IntCelP and HumPA accounted for 87.33% of total abundance (Fig. 1C). At the phylum level, dominant potential pathogens included *Proteobacteria* (88.09%), *Firmicutes* (3.49%), *Epsilonbacteraeota* (6.09%), *Chlamydiae* (2.28%), *Bacteroidetes* (0.03%), and *Actinobacteria* (0.02%) (Fig. 1B). At the genus level, dominant potential pathogens included Acinetobacter, *Stenotrophomonas*, *Arcobacter*, *Clostridium* (*Clostridium sensu stricto 1*), *Roseomonas*, and *Vibrio*, which accounted for 66.79%, 7.77%, 6.09%, 3.34%, 2.72%, and 2.00% of the total number of sequences, respectively.

### Patterns and drivers of bacterioplankton and potential pathogen communities.

**FIG 2:**
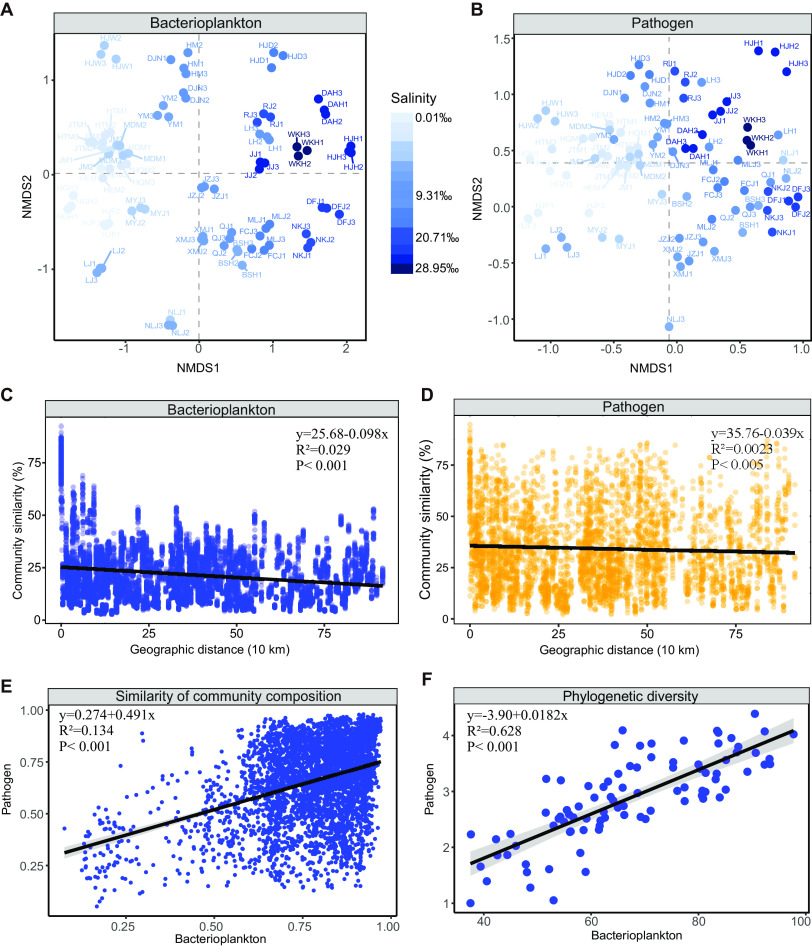
(A and B) NMDS for bacterioplankton (A) and potential pathogen communities (B) based on Bray-Curtis similarity. (C and D) Correlation between community similarity of bacterioplankton (C) and pathogens (D) and geographical distance between sites. (E and F) Relationships between bacterioplankton and pathogen diversity measured as Bray-Curtis similarity in community composition (E) and phylogenetic diversity (F).

The nonmetric multidimensional scaling (NMDS) results showed that bacterioplankton and pathogens exhibited similar biogeographic patterns. Both the bacterioplankton and pathogen communities were separated based on salinity (Fig. 2A and B). A significant distance-decay relationship between community similarity was found in the bacterioplankton (*P* < 0.001) and potential pathogen (*P* < 0.005) communities (Fig. 2C and D). Furthermore, both α diversity (Faith’s phylogenetic diversity index) and β diversity (Bray-Curtis similarity index) between the bacterioplankton and pathogens showed significant positive correlations (Fig. 2E and F), indicating similar distribution patterns.

**FIG 3:**
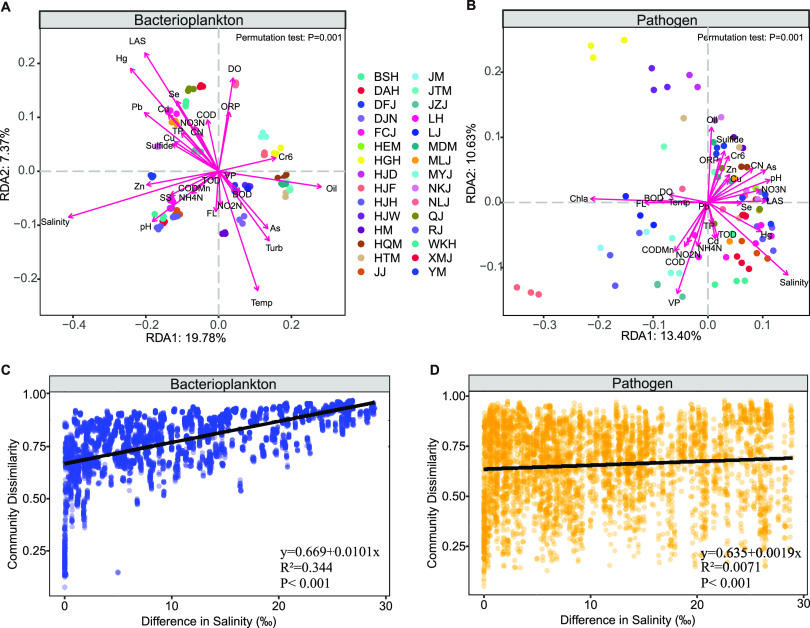
Driving factors of bacterioplankton and potential pathogens. (A and B) Redundancy analysis (RDA) showing environmental factors that influenced bacterioplankton (A) and potential pathogens (B). (C and D) Correlation between community dissimilarity of bacterioplankton (C) and pathogens (D) and salinity.

The influence of environmental parameters on the two communities was tested by redundancy analysis (RDA) (Fig. 3A and B). For the bacterioplankton communities, explanatory variables accounted for 87.2% of total variance (adjusted variation, 80.6%), with the first axis accounting for 19.78% and the second axis accounting for 15.41% of total variance. Salinity, oil, and Hg accounted for 11.84%, 8.10%, and 6.96% of total variance, respectively, with other environmental factors ranging from 0.05% to 6.31% (Pb, anionic surfactant [LAS], Zn, pH, hexavalent chromium [Cr6], turbidity [Turb], Cd, As, suspended solids [SS], sulfide, Cu, Si, temperature [Temp], total phosphorus [TP], NH_4_-N, permanganate index [CODMn], NO_3_-N, biochemical oxygen demand [BOD], cyanide [CN], dissolved oxygen [DO], chemical oxygen demand [COD], redox potential [ORP], volatile phenols [VP], total oxygen demand [TOD], fluoride [FL], and NO_2_-N) (Table 1). For the potential pathogen communities, explanatory variables accounted for 74.39% of total variance (adjusted variation, 61.36%), with the first axis accounting for 13.40% and the second axis accounting for 10.63% of total variance. Chl*a* and salinity accounted for 12.06% and 8.23% of total variance, respectively, with other environmental factors ranging from 0.37% to 6.49% (FL, pH, LAS, As, Hg, NO_3_-N, BOD, CN, DO, Se, CODMn, VP, Temp, COD, Zn, Cr6, Cu, NO_2_-N, sulfide, ORP, NH_4_-N, TOD, Cd, TP, Pb, and oil) (Table 1). Linear least-squares regression analysis showed that the bacterioplankton and potential pathogen communities were significantly correlated with salinity (Fig. 3C and D).

**TABLE 1:** Contribution of environmental factors to variations in bacterioplankton and potential pathogens explained by redundancy analysis

Environmental factor	% in:	
	Bacterioplankton	Pathogens
Salinity	11.84	8.23
Oil	8.1	0.37
Hg	6.96	5.47
Pb	5.85	0.39
LAS	5.82	5.97
Zn	5.68	2.22
pH	5.23	6.37
Cr6	4.52	2.14
Turb	3.99	NA^*a*^
Cd	3.96	0.75
As	3.88	5.93
SS	3.8	NA
Sulfide	3.65	1.69
Cu	3.48	2.11
Se	3.31	3.44
Temp	3.09	2.68
TP	2.94	0.42
NH_4_N	2.11	1.09
CODMn	2.04	3.41
NO_3_N	1.92	5.35
BOD	1.7	5
CN	1.53	4.60
DO	1.15	3.97
COD	0.88	2.39
ORP	0.85	1.25
VP	0.84	3.15
TOD	0.53	0.97
FL	0.22	6.49
NO_2_N	0.13	2.09
Chl*a*	NA	12.06

a NA, not applicable.

**FIG 4:**
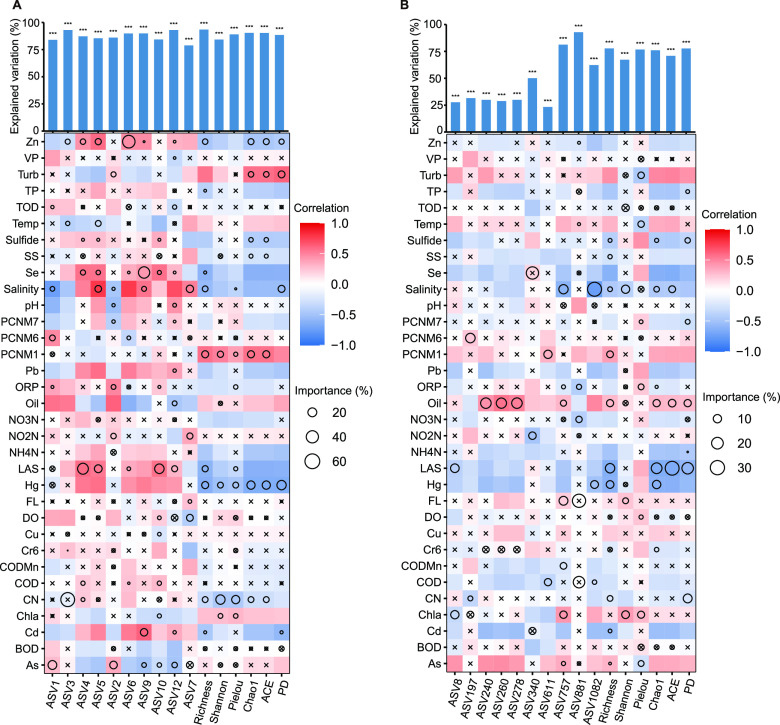
Contribution of environmental variables to the top 10 ASVs and α diversity of bacterioplankton (A) and potential pathogens (B) by random forest models and correlation. The size of the circle indicates the importance of the variable. Colors represent Spearman's correlation, and “x” indicates *P* > 0.05.

The contributions of environmental factors to α diversity and typical ASVs (top 10 most abundant) were determined based on correlation and random forest models. These measured factors explained 83.57% (62.57% to 92.41%) (Table 2) of variation in bacterioplankton and 53.99% (21.50% to 91.89%) (Table 2) of variation in potential pathogen communities in the subtropical estuaries (Fig. 4A and B). These results, combined with those from RDA (Fig. 3A and B), indicated that both the bacterioplankton and potential pathogen communities responded sensitively to local conditions in the estuaries, but potential pathogens were less influenced. In addition, the bacterioplankton and potential pathogen communities were markedly affected by both heavy metals and inorganic pollutants (Fig. 4A and B). Specifically, oil explained variation in the top 10 ASVs and α diversity, and Hg explained variation in α diversity of the pathogen communities.

**TABLE 2:** Correlations of α diversity and typical ASVs (the top 10 most abundant) with environmental factors based on correlation and random forest model

Bacterioplankton				Pathogens			
ASV and index	*R* ^2^	Adjusted *R*^2^	*P* value	ASV and index	*R* ^2^	Adjusted *R*^2^	*P* value
ASVs							
ASV1	0.8155	0.7750	1.43E-20	ASV8	0.2766	0.2600	7.63E-07
ASV3	0.9301	0.9241	1.28E-44	ASV197	0.3150	0.2911	3.70E-07
ASV4	0.9114	0.8935	9.67E-33	ASV240	0.2991	0.2830	1.93E-07
ASV5	0.8537	0.8352	7.36E-29	ASV260	0.2881	0.2718	3.80E-07
ASV2	0.8776	0.8528	1.21E-27	ASV278	0.2989	0.2828	1.96E-07
ASV6	0.9018	0.8865	9.70E-34	ASV340	0.5010	0.4836	5.56E-13
ASV9	0.8987	0.8873	4.91E-36	ASV611	0.2326	0.2150	9.94E-06
ASV10	0.8003	0.7721	7.34E-23	ASV757	0.8128	0.7836	3.60E-23
ASV12	0.6510	0.6257	4.29E-17	ASV881	0.9280	0.9189	6.99E-41
ASV7	0.8023	0.7772	8.63E-24	ASV1082	0.6231	0.6006	1.71E-16
Indices							
Richness	0.9026	0.8888	8.69E-35	Richness	0.7780	0.7434	2.08E-20
Shannon	0.8763	0.8570	6.20E-30	Shannon	0.6722	0.6260	8.50E-15
Pielou	0.8594	0.8396	1.13E-28	Pielou	0.7672	0.7237	2.58E-18
Chao 1	0.8967	0.8850	1.08E-35	Chao 1	0.7600	0.7262	7.57E-20
ACE	0.8953	0.8835	1.83E-35	ACE	0.7083	0.6834	1.79E-19
PD	0.8070	0.7880	7.91E-26	PD	0.7770	0.7456	4.75E-21

### Co-occurrence relationship between bacterioplankton and potential pathogen communities.

**FIG 5:**
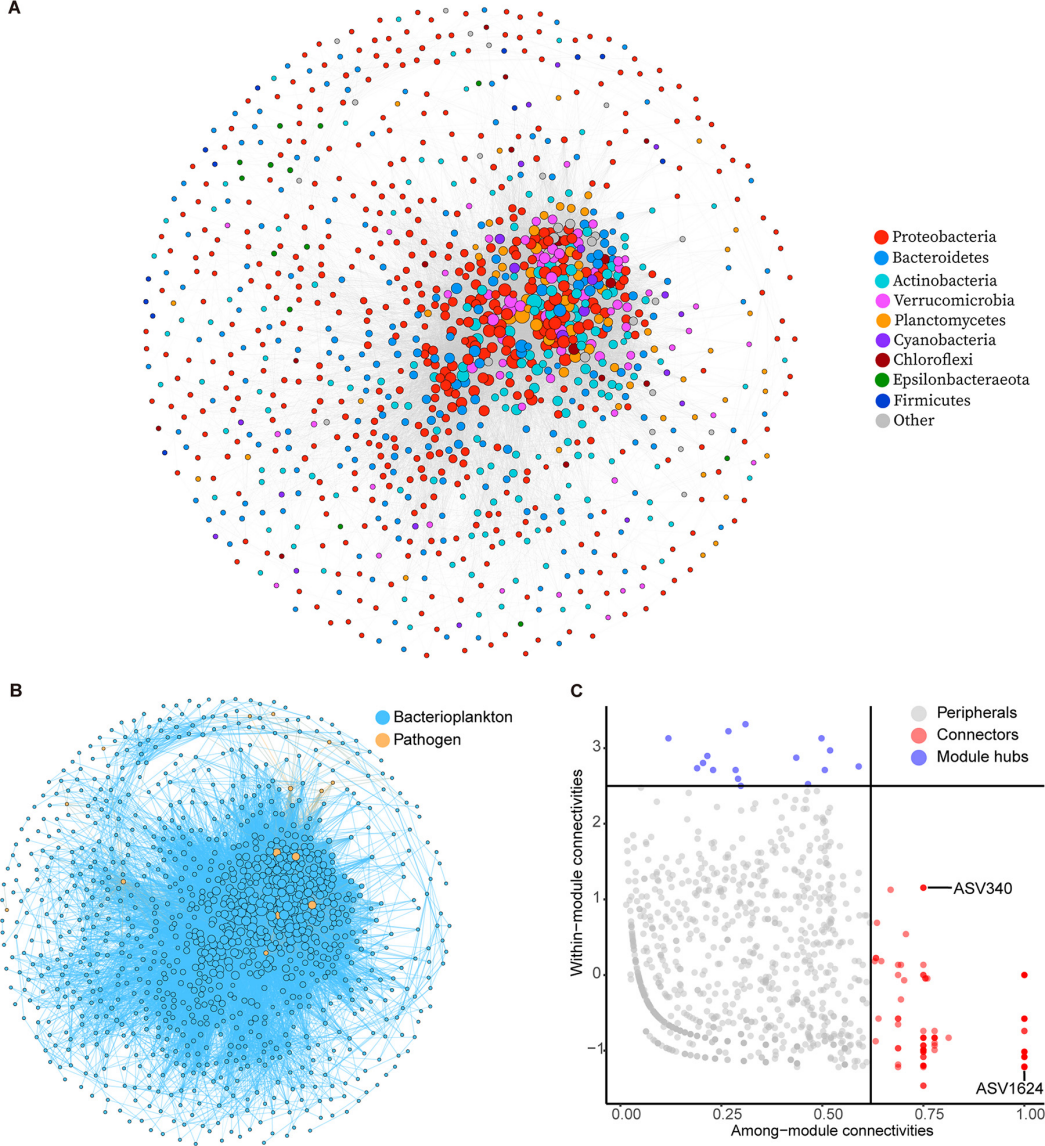
(A) Co-occurring network colored by phylum level; (B) co-occurring network colored by bacterioplankton and potential pathogen communities; (C) Zi-Pi plot showing the distribution of ASVs.

The co-occurrence network consisted of 1,077 nodes and 34,653 edges (Fig. 5A and B and Table 3), in which 1.39% of nodes were potential pathogenic bacteria (Fig. 5B). Most edges of the network (86.52%) were positive, indicating a positive symbiotic relationship for most of the community. The nine main phyla in the network were *Proteobacteria* (44.66%), *Bacteroidetes* (19.41%), *Actinobacteria* (15.13%), *Verrucomicrobia* (6.22%), *Planctomycetes* (6.13%), *Cyanobacteria* (1.95%), *Chloroflexi* (1.39%), *Epsilonbacteraeota* (1.21%), and *Firmicutes* (1.11%) (Fig. 5A).

**TABLE 3:** Parameters of correlation networks for bacterioplankton and pathogen communities in the 30 estuaries

Topological parameter	Value for parameter
No. of nodes	1,077
No. of edges	34,653
No. of positive network edges	29,982
Avg °	64.351
Network diam	10
Avg path length	3.031
Density	0.06
Modularity	0.534
Avg clustering coefficient	0.578

Zi-Pi analysis was used to infer the topological role of different nodes in the network. Results showed that 918 (85.24%) nodes were peripherals, with links inside their modules. The network contained 16 (1.49%) module hubs and 143 (13.28%) connectors (Fig. 5C), but there were no network hubs. Of note, two connectors (ASV340 and ASV1624) were pathogenic bacteria. ASV340 belongs to Clostridium perfringens (phylum *Firmicutes*), and ASV1624 belongs to Brevundimonas diminuta (phylum *Proteobacteria*).

### Assembly process of bacterioplankton and potential pathogen communities.

**FIG 6:**
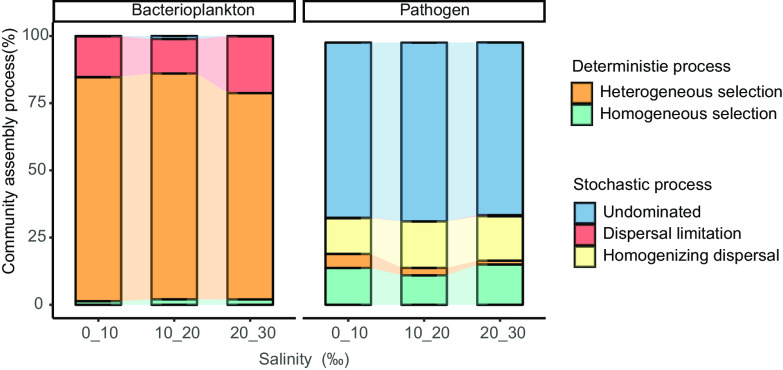
Contribution of different ecological processes to the composition of bacterioplankton and potential pathogen communities.

The β nearest-taxon index (βNTI) values of most bacterioplankton were greater than 2 or less than −2 (83.5%) (Fig. 6), indicating that deterministic processes played a greater role in the assembly of bacterioplankton communities than stochastic processes. Among the deterministic processes, heterogeneous selection (81.9%) (Fig. 6) was the most important for bacterioplankton community assembly. For potential pathogenic bacteria, only 17.5% of βNTI values were greater than 2 or less than −2, with 82.5% of values between −2 and 2, thus indicating an increase in the influence of stochastic processes in community assembly. Among the stochastic processes, ecological drift (undominated effect, 65.2%) and homogeneous dispersal (14.7%) showed the highest contribution to community assembly, far greater than that for bacterioplankton (undominated effect, 0.30%; homogeneous dispersal, 0.05%). The different salinity groups showed similar results (Fig. 6).

## DISCUSSION

Estuarine ecosystems are nutrient rich and highly productive. Bacterioplankton play an important role in ecosystem functions and services (34–36), while potential pathogens can cause diseases in aquatic organisms and humans (11–13). Pathogenic populations are dominated by microorganisms (37, 38) that are associated with anthropogenic contamination and public health risk (39, 40). However, few studies have compared the assembly processes of the bacterioplankton and potential pathogen communities. We examined the spatial variation, driving factors, and assembly mechanisms of the bacterioplankton and potential pathogen communities in a large subtropical estuarine ecosystem in southern China.

### Diversity and geographic patterns of bacterial communities and potential pathogens.

A total of 41 phyla were detected in the bacterioplankton community, with 6 phyla constituting potential pathogens. *Proteobacteria* accounted for 37.74% of the total bacterioplankton community and 88.09% of the potential pathogen community. The bacterioplankton composition is similar to those reported in previous studies in the Bohai Bay and Pearl River estuaries in China and several global estuaries (33, 41, 42). *Proteobacteria* is the most common bacterial group found in the marine environment (43). The increase in relative abundance of *Proteobacteria* under changing physical conditions may be related to the copiotrophic lifestyle of the population, whereby the population responds quickly to environmental disturbance and becomes a dominant member of the community (44). The prevalence of *Proteobacteria* may also be due to freshwater input and runoff from soils (45, 46).

High-throughput sequencing can provide a broader spectrum of pathogenic bacteria and more comprehensive information on risk assessment of potential pathogens than traditional methods: i.e., PCR, quantitative PCR, and microarrays (47–50). In this study, 31 pathogenic genera (186 ASVs) were identified. These pathogens can infect not only humans but also fish and plants. The dominant genera of pathogenic bacteria were Acinetobacter (66.79%), *Stenotrophomonas* (7.77%), and *Arcobacter* (6.09%). Of note, the dominant bacterium ASV8 (Acinetobacter lwoffii) was detected in all estuaries. Acinetobacter bacteria are human pathogens that can cause various infections, including gastritis, pneumonia, and blood circulation infections (51). Due to their remarkable ability to survive and spread in the environment, these bacteria have also become a major cause of hospital-acquired infections (52). Notably, Acinetobacter lwoffii, which is often found in soil, water, dry environments, and food, can survive even after exposure to commonly used disinfectants (53). As we used FAPROTAX, which relies on literature and cultured microbial databases, the risk of pathogenic bacteria in estuarine ecosystems may be underestimated (54, 55). Considering the economic and ecological significance of estuaries, we recommend strengthening more comprehensive early warning and monitoring programs to better assess the health risks of potential pathogens to humans and other animals in these ecosystems.

An estuary is the area in which freshwater and saltwater mix, and the different natures of the two kinds of water produce strong dynamic changes in environmental characteristics: e.g., salinity, hydrodynamics, and nutrient cycling (28, 56). In this study, clear biogeographic patterns were observed in the bacterioplankton and potential pathogen communities with salinity gradients. The results were consistent with previous studies on bacterioplankton communities in the coastal waters of China (57, 58), the Baltic Sea (27), and river-to-ocean gradients in Columbia (59). Microbes are strongly pressured or filtered by changes in environmental factors (60). Salinity fluctuations can strongly select for some bacteria and create distinct ecological niches (28). Only a few freshwater bacteria can synthesize different stress proteins to maintain metabolic activity in marine environments (36), thus limiting the access of most freshwater bacteria to estuaries. Cissoko et al. (61) demonstrated that the production of freshwater bacteria sharply declines after the addition of nutrient-rich seawater. In addition, salinity may control the distribution of bacterioplankton due to different ionic concentrations in seawater and freshwater (62), leading to the evolutionary separation of marine and freshwater bacterioplankton taxa with different adaptations to salinity (63, 64).

### Bacterioplankton and potential pathogen co-occurrence network.

The co-occurrence network showed a close relationship between bacterioplankton and potential pathogens, with two connectors (i.e., ASV340 [Clostridium perfringens] and ASV1624 [Brevundimonas diminuta]) identified as potential pathogens. *Clostridium* bacteria are strongly associated with sources of swine feces in surface and drainage waters (65). Clostridium perfringens is a Gram-positive anaerobic spore-forming bacillus widely found in soil and wastewater. It can cause food poisoning and necrotizing enteritis in humans, while in animals, it can produce toxins and enzymes, causing various symptoms (66). Brevundimonas diminuta is an opportunistic pathogen that can cause infections in humans (67). Given the pivotal roles these pathogens played in the topological structure of our co-occurrence network, assessment of their potential health risks to humans and animals and their potential impacts on microbial community structural and functional stability is critical. Previous research has shown that pathogens largely shape the structure of the microbiota (68). For example, the presence of Ralstonia solanacearum disrupts the bacterial rhizosphere microbiome during invasion (69). Furthermore, pathogenic bacteria compete with other taxa for resources and space. For instance, Pseudomonas aeruginosa increases its chances of colonizing newly available spaces by producing at least two molecules, such as rhamnolipid and the fatty acid *cis*-2-decenoic acid (70). Although co-occurrence networks can indeed identify putative interactions between microorganisms in the environment, correlation-based approaches are inherently limited when it comes to ecological interaction inferences (71). Spurious correlations in data transformation, potential confounding environmental variables, noise, and bias can lead to erroneous conclusions about species associations (71, 72). Thus, further verification is required.

### Community assembly mechanisms of bacterioplankton and potential pathogen.

Deterministic and stochastic processes are important factors governing microbial community assembly in aquatic environments (73). Due to the highly dynamic and complex environment of estuaries, strong pressure and filtering by changing environmental factors subject communities to deterministic processes (60), while dispersal and drift leave communities in stochastic dominance (74). Interestingly, two contrasting processes were observed in the bacterioplankton and pathogen communities, with deterministically dominated bacterioplankton assembly and stochastically dominated pathogen assembly. Heterogeneous selection (81.9%) was the most important process in bacterioplankton assembly, whereas ecological drift (undominated effect, 65.2%) was the most important process in potential pathogen assembly. In estuarine ecosystems, the harshness of ecological filters can increase the relative contribution of deterministic processes by influencing the adaptation of microbial communities (33). In particular, fluctuations in salt concentration can affect the energy consumption and metabolic pathways of microorganisms (75). Here, the strong effects of environmental variables on bacterioplankton suggest that environmental conditions may select microbial taxa from seed banks via special niches (57), with only well-adapted species able to survive.

Relative to bacterioplankton, stochastic processes dominated potential pathogen community assembly (indicated by βNTI in Fig. 6), similar to a previous study in the Pearl River (50). Simple attachment and spore survival strategies increase the environmental tolerance of pathogens, thereby increasing the likelihood of transmission (14, 76, 77). The dominance of stochastic processes in the potential pathogen community may be related to the ecological effects of pollution in the estuary (78–80), including urban runoff, wastewater discharge, wastewater treatment plant effluent, and concentrated animal feeding operations (17, 18). The resulting discharge can enter rivers and estuaries through various point and diffusion sources, thereby facilitating the dispersal and emergence of pathogens (81), leading to stochastically induced pathogen community assembly. The higher contribution of ecological drift to the potential pathogen community may be partially explained by differences in population size between bacterioplankton and potential pathogens (82), with smaller populations generally more impacted by ecological drift (83, 84).

### Impacts of human activities on estuarine bacterioplankton communities.

Exogenous pollutants, including nutrients and heavy metals, are important influencing factors in shaping bacterioplankton communities (85, 86). Previous studies have reported that higher levels of eutrophication and heavy metals in aquatic environments can lead to *Proteobacteria* dominance (86, 87). Species of *Proteobacteria* appear to be more tolerant to heavy metal contamination (88) and can use organic matter as an energy source (85). In the present study, the top 10 ASVs and α diversity of the pathogen community were highly correlated with oil and Hg. Runoff from estuaries and shipping activities may be potential sources of oil pollution (89). Polycyclic aromatic hydrocarbons from oil can enter the aquatic food chain through accumulation in invertebrates, causing harm to aquatic organisms and humans (90). The dominant growth strategy of mercury-stressed culturable bacterial communities shifts to rapidly growing individuals, leading to the emergence of new dominant species (91). Due to differences in the environmental plasticity of species, the ecological responses of microbial communities to pollutants may also differ widely (92), leading to the potential proliferation of tolerant or resistant populations (93). Edwards et al. (94) reported that changes in Chl*a* can predict eutrophication in coastal waters. We also found a significant correlation between Chl*a* and the pathogen community. Pathogens may enter estuaries through diverse sources, including urban runoff, wastewater treatment plant outflow, wild and domestic animal excrement, and agricultural sewage (18). Such discharge is often rich in nutrients and pathogens, which may help explain the significant association between Chl*a* and the pathogen community. Excess organic compounds in the aquatic environment can also increase virulence in pathogenic bacteria, leading to increases in the pathogenic potential of bacterial communities (95). Our study showed that the pathogen community was less sensitive to environmental factors but more likely to disperse (Fig. 3, 4, and 6); thus, the risk of effluents entering the aquatic environment should be considered and effectively treated.

### Conclusions.

In this study, amplicon sequencing and multivariate statistical analysis were used to explore the diversity of the bacterioplankton and potential pathogen communities in subtropical estuaries. High-throughput sequencing can provide broad and comprehensive information on bacterioplankton and pathogenic bacteria, thus benefiting the application of the metacommunity concept to gain deep insights into their ecological patterns. Our results revealed that the bacterioplankton and pathogen communities showed similar biogeographic patterns but were shaped by contrasting community assembly processes. These findings should advance our understanding of the biogeographic patterns and community assembly mechanisms of bacterioplankton and pathogens. This study has important implications for early warning and risk assessment of potential pathogenic bacteria, as well as management practices in estuarine ecosystems.

## MATERIALS AND METHODS

### Study area and sample collection.

**FIG 7:**
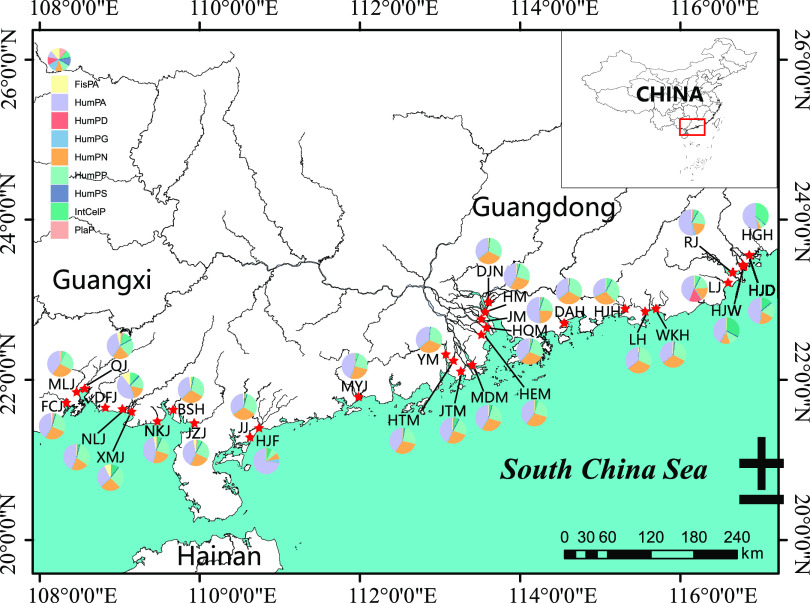
Sampling sites and 30 estuaries in the study area. A pie chart shows the composition of potential pathogenic groups at each sampling point. (BSH, river estuary of Baishahe; DFJ, river estuary of Dafengjiang; FCJ, river estuary of Fangchengjiang; MLJ, river estuary of Maolingjiang; NKJ, river estuary of Nankangjiang; NLJ, river estuary of Nanliujiang; QJ, river estuary of Qinjiang; XMJ, river estuary of Ximenjiang). The following are abbreviations associated with 22 estuaries in Guangdong Province: DAH, river estuary of Danaohe; DJN, river estuary of Dongjiangnan; HEM, Hengmen mouth; HGH, river estuary of Huanggang; HJD, river estuary of Hanjiangdong; HJF, river estuary of Huangjiangfengonghe; HJH, river estuary of Huangjianghe; HJW, river estuary of Hanjiangwaisha; HM, Humen mouth; HQM, Hongqimen mouth; HTM, Hutiaomen mouth; JJ, river estuary of Jianjiang; JM, JiaoMen mouth; JTM, Jitimen mouth; JZJ, river estuary of Jiuzhoujiang; LH, river estuary of Luohe; LJ, river estuary of Lianjiang; MDM, Modaomen mouth; MYJ, river estuary of Moyangjiang; RJ, river estuary of Rongjiang; WKH, river estuary of Wukanhe; YM, Yamen mouth.

In the winter of 2018, surface water samples (depth of 0.5 m) were collected from 30 estuaries across Guangxi Zhuang Autonomous Region and Guangdong Province in China (Fig. 7). At each estuarine site, three replicate samples were collected 30 to 50 m apart using a 2.5-L disinfection sampler (Shuitiandi Instrument, Wuhan, China). To process the samples, 500 mL of water was filtered within 4 to 8 h using a 0.22-μm-pore polycarbonate membrane (Millipore Corporation, Billerica, MA, USA). Immediately after filtration, the membrane was cooled in liquid nitrogen and stored at −80°C for DNA extraction.

### Environmental variables.

Physical hydrological parameters, including salinity, pH, temperature (Temp), dissolved oxygen (DO), and redox potential (ORP), were measured using a YSI ProPlus (YSI, Inc., OH, USA). Turbidity (Turb) was measured with a portable turbidimeter (1900C; Hach, Inc., CO, USA). Suspended solids (SS) and chlorophyll *a* (Chl*a*) were determined in the laboratory using standard methods (96). Five nutritional indices (nitrite [NO_2_N], nitrate [NO_3_N], ammonia nitrogen [NH_4_N], and total phosphorus [TP]), eight heavy metals (Cu, Zn, Se, As, Hg, Pb, Cd, and hexavalent chromium [Cr6]), and 10 bioindicators (biochemical oxygen demand [BOD], total oxygen demand [TOD], chemical oxygen demand [COD], sulfide, fluoride [FL], cyanide [CN], volatile phenols [VP], oil, permanganate index [CODMn], and anionic surfactant [LAS]) were obtained from the Guangdong Province (http://gdee.gd.gov.cn/) and Guangxi Zhuang Autonomous Region (http://sthjt.gxzf.gov.cn/) Ecological Environment Department.

### DNA extraction and sequencing.

Total microbial DNA was extracted using a HiPure Soil DNA kit (Magen, Guangzhou, China). The 16S V3-V4 hypervariable region was amplified by PCR with the 341F (CCTACGGGNGGCWGCAG)/806R (GGACTACHVGGGTATCTAAT) primer set (97). Amplicons were sequenced using the Illumina Hiseq2500 platform with PE250 mode at Gene Denovo (Guangzhou, China). A DADA2 denoising pipeline was used to obtain amplicon sequence variants (ASVs) and construct an ASV abundance table using QIIME2 v2021.4 (98). ASVs were taxonomically allocated using the QIIME2 q2-feature-classifier plugin (99) based on the SILVA v.132 database recommended by Functional Annotation of Prokaryotic Taxa (FAPROTAX) (100). To improve data analysis efficiency, low-abundance operational taxonomic units (OTUs) (<10) were abandoned. Finally, a total of 6,737,136 high-quality sequences were obtained. The ASV abundance table was rarefied to the lowest number of tags (32,542).

### FAPROTAX annotations.

FAPROTAX is a commonly applied prediction method that uses 16S rRNA gene sequencing to extrapolate microbial classification to putative functions based on literature and cultured microbial databases (54). ASVs belonging to at least one of the following functions were assigned as potential pathogens: i.e., intracellular parasites (IntCelP), human potential pathogens—all (HumPA), human potential pathogens—pneumonia (HumPP), human potential pathogens—nosocomial (HumPN), plant potential pathogens (PlaP), human potential pathogens—diarrhea (HumPD), human potential pathogens—septicemia (HumPS), fish parasites (FisPA), and human potential pathogens—gastroenteritis (HumPG).

### Statistical analysis.

Nonmetric multidimensional scaling (NMDS) was used to display differences in ASV-level community composition along the estuaries. Redundancy analysis (RDA) and correlation analysis were carried out to illustrate the influence of environmental attributes on community structure using the forward selection method. If significance reached *P* > 0.05 or selection criteria (*R*^2^) did not improve when additional variables were added, forward selection was stopped. NMDS and RDA analyses were done using the 2.6-2 version of the vegan package (101). A set of spatial variables (e.g., PCNM1, PCNM6, and PCNM7) was calculated using the principal coordinates of neighbor matrices (102). Following Stegen et al. (84), null-model analysis was used to decipher the assembly mechanisms underlying the bacterioplankton and pathogen communities, calculated via the β nearest-taxon index (βNTI) and Raup-Crick (RC) metric using the 1.8-2 version of the “picante” package (103). βNTI values of less than −2 or greater than 2 indicated homogeneous and heterogeneous selection, respectively. For βNTI values greater than −2 and less than 2, RC absolute values of <0.95 indicated undominated effects, RC values of >0.95 indicated dispersal limitations, and RC values of less than −0.95 suggested homogeneous dispersal (26).

Co-occurrence among ASVs was studied using network analysis by Spearman correlations. To simplify the network for better visualization, ASVs occurring in at least 20% of samples were reserved. Spearman dependencies were calculated using the 4.3-0 version of the “Hmisc” package (104). Network visualization and topological analysis were carried out using the v.0.9.2 version of Gephi software (|*r*| > 0.60; *P* < 0.05) (105). To identify key populations that are essential, nodes were divided into four categories according to among-module connectivity (Pi) and within-module connectivity (Zi): i.e., module hubs (Pi < 0.62; Zi > 2.5), connectors (Pi > 0.62; Zi < 2.5), network hubs (Pi > 0.62; Zi > 2.5), and peripherals (Pi < 0.62; Zi < 2.5) (106).

### Data availability.

Sequencing data have been deposited in the NCBI SRA under accession no. PRJNA730095.
